# Editorial: Editors’ showcase 2022: insights in molecular and cellular reproduction

**DOI:** 10.3389/fcell.2023.1321358

**Published:** 2023-11-13

**Authors:** Jia-Qian Ju, Rafael A. Fissore, Shao-Chen Sun

**Affiliations:** ^1^ College of Animal Science and Technology, Nanjing Agricultural University, Nanjing, China; ^2^ Department of Veterinary and Animal Sciences, University of Massachusetts Amherst, Amherst, MA, United States

**Keywords:** folliculogenesis, oocyte, spermatogenesis, fertilization, embryo, placenta

## Introduction

Germ cells, which give rise to sperm and eggs, undergo meiosis during their differentiation process. Meiosis is a specialized form of cell division that results in the production of haploid cells from diploid cells. In this process, the chromosomes in the germ cells are duplicated only once, followed by two rounds of cell division. This leads to the formation of haploid cells, each containing half the number of chromosomes compared to the original diploid cell. However, after the haploid sperm and egg are formed, they come together through fertilization, wherein they fuse to create a diploid zygote. The zygote then develops into a new organism, initiating the next cycle of life.

Meiosis is also essential for sexual reproduction and the generation of genetic diversity, as crossing over between different-origin chromosomes takes places during the early stages of this process. Therefore, both molecular and cellular reproduction are fundamental processes that ensure the continuity of life and the transmission of genetic information from one generation to the next and whose underlying mechanism(s) must be understood.

This Research Topic includes 1 method, 2 reviews, and 11 original research articles, providing critical and novel insights into cell and developmental biology. Specifically, it sheds light on folliculogenesis, oocyte maturation, embryo development, spermatogenesis, sperm maturation, sperm capacitation, and fertilization.

## Folliculogenesis and oocyte maturation

Female fertility female is closely associated with several processes, including folliculogenesis and oogenesis (the development of follicles, oocytes and eggs, respectively) and oocyte maturation. Here, 4 articles introduce and discuss aspects of female fertility.

The initiation of estrous cycles in female mammals, which marks the initiation of the fertile period, is prompted by an increase in the circulating levels of gonadotropins, including the pre-ovulatory luteinizing hormone (LH). The LH surge serves as the stimulant for the onset of oocyte maturation, follicle cell luteinization, increased follicular vascularity, and breakdown of the follicle wall that leads to ovulation (Robker et al., 2018). According to Hessock et al., the LH surge results in an increased availability of amino acids and metabolites for cumulus and granulosa cells, which are utilized for energy production and also transferred to the oocyte to support meiotic maturation. Furthermore, at a later stage, these metabolites may also be utilized by the ovulatory follicle for protein production.

Oogenesis is the complex process of female gamete (oocyte) growth and maturation that occurs in a regulated fashion and in waves in the ovaries and continues throughout their reproductive lifespan. PGs (prostaglandins) have been reported to regulate almost every aspect of female reproduction. For example, loss of PG synthesis blocks mammalian follicle maturation and ovulation (Tootle, 2013). According to Talbot et al., PGs play a crucial role during oogenesis in carefully regulating the level and forms of nuclear actin. This regulation is necessary to control nucleolar activity required for the production of fertilization-competent oocytes. In addition, microtubule movement plays an important role in mRNA localization and modeling in *Drosophila* oocytes (Goldman and Gonsalvez, 2017). Neiswender et al. recently identified the *Drosophila* ortholog of TTC1 (dTtc1) as an interacting partner of Egalitarian, an RNA adaptor of the Dynein motor, and showed that the depletion of dTtc1 leads to defective oogenesis in *Drosophila* (fruit flies), resulting in the absence of mature eggs. Further examination revealed that the egg chambers without dTtc1 displayed a distinct phenotype, characterized by severely swollen mitochondria. Furthermore, Oocyte quality and its genetic integrity play a crucial role in fertility. Environmental toxins can negatively affect oocyte quality and fertility, and have recently become a cause for concern, which also threatens food safety. There is growing evidence that Azoxystrobin (AZO) is widespread in the environment and has the potential to induce developmental toxicity in animals. For example, AZO has been reported to impair neuronal migration by inducing mitochondrial deactivation and induction of apoptosis increasing (Kang et al., 2021). Gao et al. suggested that AZO is one of the most widely used fungicides in agriculture, and female exposure to AZO impairs oocyte maturation by negatively influencing a variety of cellular functions such as increasing oxidative stress and mitochondrial dysfunction, decreasing MTOC integrity and the subsequent spindle formation and chromosome alignment.

## Spermatogenesis, sperm capacitation and fertilization

Spermatogenesis is the process of sperm cell development in the testes of male organisms. It involves the production and maturation of sperm cells, also known as spermatozoa. Spermatogenesis is an extraordinarily complex process. The differentiation of spermatogonia into spermatocytes, spermatid and finally in mature spermatozoa requires the participation of non-spermatogenic cell types, hormones, paracrine factors, genes, and epigenetic regulators. Here, a total of 8 articles introduced the effects of the fertility of male.

Efficient sperm production requires the translocation of protamines from the cytoplasm to the nucleus. The Sperm-associated antigen 17 (SPAG17) is found to be expressed in testicular germ cells during the late stages of sperm development and is shown to localize to the manchette, contributing to protein trafficking (Kazarian et al., 2018). Here, Agudo et al. discovered for the first time that Spag17 was essential for normal manchette structure, protein transport, and formation of the sperm head and flagellum, in addition to its role in sperm motility. During sperm maturation, significant changes occur, including the loss of most membrane organelles and a substantial accumulation of ether glycerolipids, that is a well-conserved phenomenon across different species. These specific lipid molecules contribute to the unique composition and properties of sperm cells (Teves and Roldan, 2022). Remedios et al. reviewed the existing knowledge on the relevance of the different types of ether lipids involved in sperm production, maturation, and function, and provided a comprehensive metabolic map associated with ether-lipids and peroxisomal-related functions in sperm.

After ejaculation, sperm cells undergo a process called capacitation, which is necessary for them to become fully capable of fertilizing an egg. Targeted disruption of the soluble adenylyl cyclase (ADCY10; sAC) gene results in male-specific sterility without affecting spermatogenesis, mating behavior, or spermatozoa morphology and count; however, it dramatically impairs sperm motility and prevents capacitation (Akbari et al., 2019). Research by Ritagliati et al. demonstrate that sAC KO male mice are infertile because their sperm do not penetrate beyond the UTJ suggests that human’s sAC KO men are infertile because their sperm will not cross the cervix to enter the uterus to be able to ever reach the oviduct and the fertilization site. Because the vagina reacidifies following intercourse, in the absence of sAC, human sperm are not likely to survive long after sex. Additionally, Balbach et al. compared the metabolic changes that occur during capacitation in mouse sperm obtained from the epididymis versus ejaculated sperm. They also related these changes to human ejaculated sperm. The study revealed that capacitation induced metabolic changes in pathways that support the motility of both epididymal and ejaculated sperm. During capacitation, sperm experience a change in their motility pattern to a more vigorous, this process requires the uptake of calcium ions through the sperm-specific CatSper channel complex. This channel represents a promising target for contraception research. Recent advances have reported that the rise in intracellular Ca^2+^ concentration after exposure to an alkaline-high K^+^ solution was evaluated as an indirect measure of CatSper function in a high-throughput drug screening (Carlson et al., 2022). Here, Luque et al. proposed an alternative method to specifically evaluate CatSper opening. Their approach involves removing external free divalent cations through chelation, which then allows CatSper to efficiently conduct monovalent cations. By using this method, researchers can test various compounds to determine their ability to inhibit CatSper function in sperm. This screening process is valuable for identifying potential drugs or compounds that could target CatSper as a means of contraception or fertility control.

Fertilization occurs when a sperm cell successfully negotiates the outer protective layers of the egg and fuses with the egg’s plasma membrane. Sperm contain a single, large dense-core secretory granule (the acrosome) whose contents are released by regulated exocytosis (acrosomal exocytosis, AE) prior to fertilization. α-Synuclein, a small cytosolic protein, has emerged as an important regulator for membrane fusion (Khounlo et al., 2021). Here, Buzzatto et al. have discovered that α-Synuclein is necessary for sperm exocytosis in a post-fusion stage for expanding the fusion pores in the acrosome. In oocytes, the glycophosphatidylinositol (GPI) anchored protein Juno (Bianchi et al., 2014) and a member of the tetraspanin family, CD9 (Kaji et al., 2000), have been identified as critical for mammalian fertilization. Frolikova et al. propose the existence of Juno-CD9 complexes in specific spatially defined compartments within the microvillar regions of the mouse oolemma. This complex is believed to play a distinct functional role in sperm binding sperm to the oocyte. This research provides insights into the molecular mechanisms involved in sperm-oocyte interactions and adds to our understanding of the fertilization process. Understanding the genetic underpinnings of fertilization is essential for developing infertility treatments, contraceptive targets, understanding speciation, and mechanisms of cell-cell interactions. Maniates and Singson discussed potential experimental biases and intrinsic biological factors that hinder the discovery of genes related to fertilization. They also shed light on current strategies aimed at identifying these genes, which may lead to further advancements in understanding this complex process.

## Embryo and placenta

The embryo and placenta are two crucial components of mammalian reproduction. The embryo is the early stage of development of a multicellular organism. Fertilization results in the formation of a zygote, which undergoes a series of cell divisions called cleavage. As the cells continue to divide and differentiate, the embryo gradually forms. The placenta is an organ that develops during pregnancy in mammals and plays a critical role in supporting the growth and development of the fetus during pregnancy. It facilitates the exchange of nutrients, gases, and waste products between the mother and the developing baby, while also producing hormones and providing immune protection. Here, a total of 2 articles introduced the embryo and placenta.

Previous reports have shown that the pig extraembryonic endoderm (pXEN) cells contribute both to extraembryonic tissues including visceral yolk sac as well as embryonic gut when injected into host blastocysts, and generate live offspring when used as a nuclear donor in somatic cell nuclear transfer (SCNT) (Park et al., 2021). Based on this, Park et al. developed a method for generating primary fibroblast-like cells from pluripotent cells from embryonic outgrowths and tested the hypothesis that these fibroblast-like cells derived from the embryo serve as an ideal nuclear donor for embryo transfer procedures. They showed that embryo-derived fibroblasts (EFs) share many characteristics with fetal fibroblasts (FFs), including the spindle-shaped morphology and expression of key fibroblast-specific molecular markers. In addition, transcriptomic analysis via RNA sequencing (RNA-seq) confirmed the similarity of EFs to FFs. The EFs used as nuclear donors for SCNT resulted in enhanced *in vivo* developmental competence.

The placenta integrates an array of signals from both the mother and fetus to maintain fetal homeostasis. It performs numerous functions critical for normal fetal growth and development, including mediating nutrient and waste transfer, secreting hormones, serving as an immunological barrier, and performing xenobiotic detoxification (Díaz et al., 2014). Kelly et al. perform RNA-seq analysis at embryonic day 18.5 to identify genes differentially expressed in the placentas of obese and normal-weight dams (controls) using a mouse model of diet-induced obesity with fetal overgrowth. The data shows that maternal obesity with fetal overgrowth differentially regulates the transcriptome in male and female placentas, including genes involved in oxidative phosphorylation. They also provide evidence that the findings in mice have clinical relevance because the expression of the placental mitochondrial complex in humans was downregulated and negatively correlated with maternal pre-pregnancy BMI and birth weight in male infants.

Overall, the findings in this Research Topic provide new insights into the molecular and cellular aspects of many reproductive processes, including aspects of germ cell specification, folliculogenesis, spermatogenesis, oocyte maturation, sperm capacitation, and embryo development. The new information has broad implications for understanding fundamental biological processes, disease mechanisms, and implementing potential therapies.
